# The clinical, immunological and genetic features of 12 Chinese patients with STAT3 mutations

**DOI:** 10.1186/s13223-020-00462-w

**Published:** 2020-07-22

**Authors:** Li Lin, Ying Wang, Bijun Sun, Luyao Liu, Wenjing Ying, Wenjie Wang, Qinhua Zhou, Jia Hou, Haili Yao, Liyuan Hu, Jinqiao Sun, Xiaochuan Wang

**Affiliations:** 1 Department of Clinical Immunology, Children’s Hospital of Fudan University, 399 Wanyuan Road, Shanghai, 201102 China; 2 Department of Neonatology, Children’s Hospital of Fudan University, 399 Wanyuan Road, Shanghai, 201102 China

**Keywords:** STAT3, Hyperimmunoglobulin E syndrome, HIES, Primary immunodeficiency disease, STAT3 deficiency

## Abstract

**Background:**

Loss-of-function (LOF) mutations in signal transducer and activator of transcription 3 (STAT3) is one of the causes of STAT3 hyperimmunoglobulin E (IgE) syndrome (STAT3-HIES), while gain-of-function (GOF) mutations in STAT3 lead to immune dysregulation diseases. We retrospectively analyzed the age, common clinical symptoms, immunologic and molecular manifestations in 11 patients with LOF STAT3 mutations and 1 patient with a GOF STAT3 mutation.

**Methods:**

Twelve patients were enrolled in our study. Serum immunoglobulin measurements, lymphocyte subset detection and whole-exome sequencing were performed.

**Results:**

The median age at diagnosis of STAT3-HIES patients was 4.74 years. Eczema, recurrent respiratory infections, fevers, abscesses and *Staphylococcus aureus* infections were the classic manifestations. Elevated serum IgE levels are not always observed in conjunction with high eosinophil counts. A moderate viral DNA load was also measured in peripheral blood mononuclear cells. We noticed that c. 1144C>T was the most common mutation site, followed by c.1311C>A. Additionally, c.1311C>A and c. 1826G>C are two novel mutations. Eight patients achieved notable improvement after receiving intravenous immunoglobulin.

**Conclusion:**

We updated the current knowledge of this topic. We found an earlier median age at diagnosis, a higher survival rate, and a general lack of nonimmunological abnormalities; we also described the treatment details and novel mutations involve in STAT3-HIES and compared STAT3 LOF and GOF mutations.

## Background

Loss-of-function (LOF) mutations in the signal transducer and activator of transcription 3 (STAT3) gene constitute one of the underlying causes of the autosomal dominant (AD) hyperimmunoglobulin E (IgE) syndrome (HIES). The most current disease abbreviation is STAT3-HIES [1]. STAT3-HIES was first described as Job syndrome in 1966, and it is characterized by eczematous dermatitis, recurrent skin and pulmonary abscesses and elevated serum IgE levels [2–4]. Non-immunological abnormalities are bone abnormalities, retention of the primary teeth, specific facial features and lack of growth [5]. There is no predominant sex or ethnicity among STAT3-HIES patients [6]. *Staphylococcus aureus* and candidiasis are the most common pathogens [7]. In addition, STAT3-deficient patients are more susceptible to bacterial and fungal infections and exhibit poor control of Epstein–Barr virus (EBV) and Varicella–zoster virus (VZV) infections [8].

The use of antibiotics and the prevention of complications are recommended as the treatment strategy for STAT3-HIES patients; hematopoietic stem cell transplantation (HSCT) was found to be ineffective [9].

Gain-of-function (GOF) mutations in STAT3 are associated with multiorgan autoimmune disorders and immunodeficiency. Immunosuppressive therapy and targeted biotherapy are recommended for those patients [10].

Approximately 500 cases of STAT3-HIES have been reported worldwide, but only 50 cases were in Chinese individuals. There have only been two relatively large cohort studies and sporadic case reports in different areas of China [11, 12]. Herein, we describe a cohort of 12 Chinese patients carrying STAT3 mutations. We investigated their clinical, immunologic and genetic characteristics. Our description helps expand the spectrum of known STAT3 mutation diseases in different ethnic groups.

## Methods

### Patients

We collected data from 12 patients at the Children’s Hospital of Fudan University from July 2017 to June 2019. Written informed consent was obtained from all participants’ guardians before inclusion in the study. The study was approved by the Ethics Committee of the Children’s Hospital of Fudan University.

### Serum immunoglobulin and lymphocyte subset detection

As previously reported [13], serum IgG, IgA and IgM were determined by an automated clinical chemistry analyzer (Erba Diagnostics, Mannheim, Germany). IgG (Cat. No. 67731), IgA (Cat. No. 67746) and IgM (Cat. No. 67732) reagents were purchased from Orion Diagnostica Oy (Espoo, Finland). IgE reagent was purchased from Jing Yuan Corp. (Shanghai, China), and IgE was assessed with UniCAP (Pharmacia, Uppsala, Sweden).

Flow cytometry was performed according to our previously published protocol [14]. Briefly, staining for lymphocyte surface markers was performed after red blood cell lysis. After washing with cold phosphate buffer solution containing 2% FBS two times, 1 × 10^4^–5×10^4^ live cells were analyzed with a FACSCalibur flow cytometer (Becton–Dickinson, Franklin Lakes, NJ, USA) using Diva software (BD Biosciences). B cells, total T cells, CD4+ T cells, CD8+ T cells and CD56+/CD16+ natural killer (NK) cells were detected with the BD Multitest IMK Kit. T cell subsets were defined by anti-human CD3 (PerCP-Cy5.5), anti-CD8 (BV510), anti-CD4 (FITC; fluorescein isothiocyanate), anti-CD27 (APC; allophycocyanin), anti-CD45RA (PE-Cy7), anti-TCRαβ (PE; phycoerythrin) and anti-TCRγδ (BV421). The following B cell subsets were detected: anti-CD19 (APC), anti-human CD24 (PE), anti-CD27 (BV450), anti-CD38 (PerCP-Cy5.5) and anti-IgD (BV510) (BD Biosciences).

### Whole-exome sequencing (WES)

WES and analysis protocols were adopted for the genetic analysis. Briefly, genomic DNA was extracted from the whole blood samples of the patients and their parents. Then, genomic DNA fragments were enriched for the target region of the consensus coding sequence exons and subsequently sequenced on the HiSeq 2000 sequencer (Illumina, San Diego, CA). The raw data were mapped to the human genome reference sequence (hg19). Nucleotide changes observed in more than 5% of the aligned reads were called and reviewed with NextGENe software (SoftGenetics, State College, PA).

Mutations in STAT3 were confirmed by Sanger sequencing. DNA was extracted from the patients and their relatives. Peripheral blood mononuclear cells (PBMCs) were isolated using the RelaxGene Blood DNA System (Tiangen Biotech, Beijing, China) according to the manufacturer’s protocol. Primers were designed to span each exon, and PCR amplification of STAT3 was carried out at 94 °C for 3 min, followed by 35 cycles at 94 °C for 30 s, 60 °C for 30 s and 72 °C for 40 s. The final extension was performed at 72 °C for 10 min. The PCR product was sequenced in both directions by ABI Prism BigDye terminators.

### Statistical analysis

The data management and statistical analysis were performed with GraphPad Prism 8 software (GraphPad Software, La Jolla, Calif).

## Results

### Overview

Table 1 shows the general information of the patients. The 12 patients, including seven males and five females, were all from nonconsanguineous families. Eleven patients were diagnosed with STAT3 LOF mutations, and one patient (P12) was diagnosed with a STAT3 GOF mutation. Eleven STAT3-deficient patients were diagnosed at a median age of 4.74 years (0.5–12 years old), while the median age at symptom onset was 1.89 years (neonate-11.5 years old), with 5 infantile-onset patients (P1, P7, P8, P10 and P11). The age at symptom onset in STAT3 GOF mutation patient was 13 years. All the patients were full-term, with either cesarean section (2/12) or spontaneous (10/12) deliveries.

**Table 1:** General information of STAT3 mutation patients

	P1	P2	P3	P4	P5	P6	P7	P8	P9	P10	P11	P12
Gender	Male	Male	Female	Female	Male	Female	Male	Male	Male	Female	Male	Female
Age at symptom onset (year)	Neonate	11.5	1	1.5	0.3	0.3	Neonate	Neonate	5	Neonate	Neonate	13
Age on clinical diagnosis (year)	10	12	4.5	2.25	1.3	1.25	10	0.5	6	3.5	0.83	14
Mutation of STAT3												
cDNA	1863C>G	1311C>G	1144C>T	1145G>C	1826G>C	1144C>T	1827A>T	1144C>T	1311C>G	1909G>A	994C>A	1261G>A
Amino acid	F621L	H437Q	R382W	R382P	R609T	R382W	R609S	R382W	H437Q	V637M	H332N	G421R
Type	De novo	De novo	De novo	De novo	De novo	De novo	De novo	De novo	De novo	De novo	Paternal	De novo
Immunological abnormalities												
Fever	−	+	−	+	+	+	+	−	+	+	+	+
Eczema	+	+	+	+	+	+	+	+	+	+	+	−
Respiratory infection	+	+	+	+	+	+	+	+	+	+	+	+
Skin and pulmonary abscess	+	+	+	+	−	−	+	−	−	+	−	−
Otitis media	+	−	+	+	+	+	−	−	−	−	−	+
Thrush	−	−	+	−	+	+	−	+	+	−	−	+
Diarrhea	−	−	−	−	−	−	+	+	−	−	−	+
Enlarged lymph nodes	−	+	−	+	−	+	+	−	−	−	−	+
Hepatosplenomegaly	−	−	−	−	+	−	−	−	−	−	−	+
Non-immunological abnormalities												
Failure to growth	−	−	−	−	+	−	+	−	−	−	−	+
Skeletal abnormalities	−	−	−	−	+	−	+	−	−	−	−	−
Retention of primary teeth	+	+	−	−	−	−	+	−	−	−	−	−
Facial features	+	−	−	−	+	−	+	−	−	−	−	−
Other presentations	RDS, Fatty liver				Cryptorchidism, Laryngeal cleft, Fissured tongue	Dysfunction of liver, Pityriasis rubra pilaris, Congenital laryngeal cartilage dysplasia; CMV infection	Dysfunction of liver, Leukoma, EBV infection	ASD, Vaccinated scar, CMV infection, Agranulocytosis, Respiratory Failure, Dysfunction of liver	Fungal infection, Cavernous hemangioma, Cryptorchidism, Leukopenia, Laryngotracheal stenosis	Rhinitis, EBV infection	Cholestasis	Rhinitis, Autoimmune hemolytic anemia, Myasthenia, Diabetes, Alopecia, Delayed pubertal development, EBV infection
NIH scores	53	47	62	49	61	41	62	27	40	45	21	ND
Eosinophils (/μl)	780	5860	689	720	2650	4000	1350	589	220	470	570	10

*ND* Not determined, *RDS* respiratory distress syndrome, *ASD* atrial septal defect, *CMV* cytomegalovirus, *EBV* Epstein–Barr virus

### Infectious complications of 11 STAT3 LOF patients

**Fig. 1:**
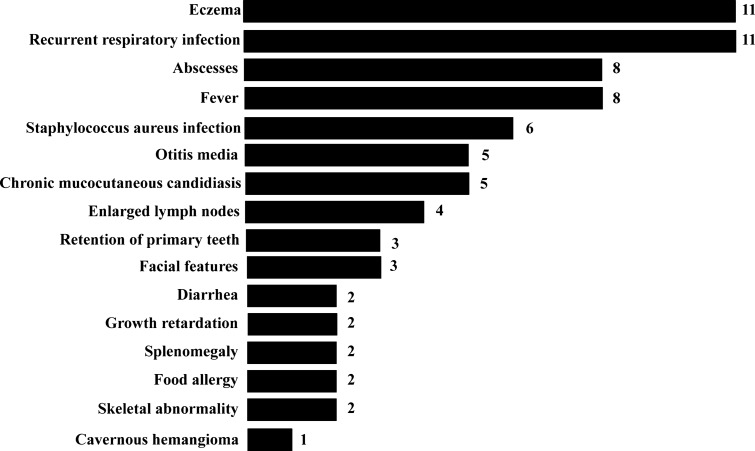
A variety of clinical manifestations of 11 patients carrying loss-of-function (LOF) mutations in STAT3. The number is the number of STAT3 LOF mutation patients, and the Y-axis shows the different disease symptoms

All patients suffered from eczema, especially in the facial and scalp areas. More seriously, some eczema spread from the scalp to the limbs. Recurrent respiratory infections were the most common manifestation in our patients (11/11). Moreover, infections in other systems were also observed, such as otitis media in 5 (P1, P3, P4, P5 and P6), rhinitis in 1 (P10) and diarrhea in 2 (P7 and P8). Rotavirus was detected in P8. *Staphylococcus aureus* infection is one of the noteworthy characteristics of STAT3-deficient patients. Almost half of the patients (P2, P3, P4, P5, P7 and P9) had *S. aureus* pneumonia that was confirmed by either sputum, blood, or bronchoalveolar lavage fluid (BALF) culture. Abscesses occurred in 8 patients in different body parts: lungs in 5 (P2, P3, P7, P9 and P10), the scalp in 4 (P2, P4, P7 and P10), the abdomen in 1 (P7) and the gluteal region in 1 (P1). Moreover, P7 and P10 underwent partial lung lobectomy. Approximately half of our patients (5/11. P3, P5, P6, P8 and P9) had marked chronic mucocutaneous candidiasis (CMC) verified by microscopic examination fungal cultures. Eight patients (8/11. P2, P4, P5, P6, P7, P9, P10 and P11) had a fever (Fig. 1).

EBV-DNA was measured in the PBMCs in 2 out of the 11 patients (P7 and P10), and the viral loads were 9.00E+03 and 2.60E+03, respectively. Cytomegalovirus (CMV)-DNA was also detected in blood, urine and BALF samples in two patients (P6 and P8) with viral loads of 8.69E+04 and 2.00E+05, respectively.

### Immunological presentation in 11 STAT3 LOF mutation patients

The eosinophil count was elevated to different degrees (470–5860 cells/μL, reference range: 30–500 cells/μL) (Table 1). Elevated serum IgE is considered one of the most prominent characteristics in STAT3-deficient patients. Except for P8 (67.2 KU/L) and P11 (43.87 KU/L), the other patients had high serum IgE levels ranging from 1841.29 to 17310.4 KU/L (the average reference value range was < 100 KU/L). The IgG, IgA and IgM levels remained approximately normal (Table 2).

**Table 2:** Lymphocytes subsets and immunoglobulin of STAT3 mutation patients

	P1	P2	P3	P4	P5	P6	P7	P8	P9	P10	P11	P12
Lymphocytes subsets												
Total T cells (cells/μL)	2418.05↑ (1325–2276)	1640.43 (1325–2276)	2210.10 (1480–2847)	4180.60↑ (1775–3953)	3722.60 (1794–4247)	2748.03 (1775–3953)	2198.60 (1325–2276)	2484.20 (2187–6352)	1704.50 (1424–2664)	1524.7 (1775–3953)	3343.50 (2187–6352)	665.20↓ (1169–2071)
Total T cells (%)	79.79↑ (57.10–73.43)	72.59 (57.10–73.43)	72.99 (59.50–75.56)	71.41 (53.37–71.91)	65.59 (53.88–72.87)	64.26 (53.37–71.91)	77.67↑ (57.10–73.43)	60.47 (55.32–73.11)	74.92 (60.05–74.08)	59.09 (53.37–71.91)	60.88 (55.32–73.11)	79.77↑ (61.29–73.13)
CD4 T cells (cells/μL)	935.32 (531–1110)	859.30 (531–1110)	1537.59 (767–1592)	2784.37↑ (948–2477)	2073.18 (902–2253)	1900.82 (948–2477)	1346.17↑ (531–1110)	1403.91 (1125–3768)	936.74 (686–1358)	833.11 (948 –2477)	1812.96 (1125–3768)	347.24↓ (554–1109)
CD4 T cells (%)	30.98 (24.00–38.72)	37.96 (24.00–38.72)	50.78↑ (28.49–41.07)	47.56↑ (26.19–45.48)	37.38 (24.08–42.52)	45.86 (26.19–45.48)	47.56↑ (24.00–38.72)	34.17 (28.17–47.74)	41.17 (26.17–40.76)	32.29 (26.19–45.48)	33.01 (28.17–47.74)	39.03 (26.36–40.90)
CD8 T cells (cells/μL)	1133.59 (480–1112)	579.86 (480–1112)	620.54 (553–1127)	1291.87 (531–1521)	1480.57 (580–1735)	766.79 (531–1521)	757.71 (480–1112)	934.42 (686–2278)	521.27 (518–1125)	614.41 (531–1521)	1299.38 (686–2278)	265.24↓ (423–900)
CD8 T cells (%)	37.26↑ (21.01–33.94)	25.29 (21.01–33.94)	20.49 (19.70–32.04)	22.07 (16.29–29.88)	25.38 (19.00–32.51)	16.60 (16.29–29.88)	26.77 (21.01–33.94)	22.74 (15.88–31.48)	22.91 (19.68–34.06)	23.81 (16.29–29.88)	23.66 (15.88–31.48)	35.13↑ (20.99–33.73)
Total B cells (cells/μL)	334.16 (216–536)	228.69 (216–536)	719.88 (303.52–777.25)	1238.04 (537.11–1464.39)	1500.17↑ (461–1456)	1320.16 (537.11–1464.39)	560.52↑ (216–536)	957.21 (916–1832)	366.17 (280–623)	801.72 (537.11–1464.39)	1732.5 (916–1832)	116.07↓ (176.56–415.64)
Total B cells(%)	11.23 (9.19–19.48)	11.96 (9.19–19.48)	23.78↑ (10.46–21.77)	21.15 (13.93–30.49)	26.67 (13.23–26.39)	28.15 (13.93–30.49)	19.80 (9.19–19.48)	23.30 (17.20–29.71)	16.09 (10.21–20.12)	31.07 (13.93–30.49)	31.55↑ (17.20–29.71)	16.24 (7.73–16.84)
NK cells (cells/μL)	245.275 (246–792)	306.80 (246–792)	64.19↓ (227–668)	373.53 (241–978)	386.41 (270–1053)	329.45 (241–978)	17.08↓ (246–792)	597.21 (306–896)	190.56↓ (258–727)	208.92↓ (241–978)	384.33 (306–896)	32.05↓ (232–789)
NK cells(%)	8.23 (10.01–26.98)	14.31 (10.01–26.98)	2.12↓ (7.83–20.99)	6.38↓ (6.53–22.24)	6.53↓ (7.21–20.90)	6.03↓ (6.53–22.24)	0.60↓ (10.01–26.98)	14.53 (5.67–15.90)	8.38↓ (9.00–22.24)	8.1 (6.53–22.24)	7.00 (5.67–15.90)	2.85↓ (11.43–27.57)
CD4/CD8	0.84 (0.81–1.66)	1.53 (0.81–1.66)	2.48↑ (1.02–2.05)	2.16 (1.05–2.53)	1.52 (0.90–2.13)	2.85 (1.05–2.53)	1.78↑ (0.81–1.66)	1.50 (0.93–2.52)	1.80 (0.87–1.94)	1.36 (1.05–2.53)	1.4 (0.93–2.52)	1.32 (0.85–1.76)
Immunoglobulin												
IgG (g/L)	13.90	9.10	17.1↑	9.70	9.90	9.20	14.00	7.30	12.60	11.90	7.00	14.50
IgA (g/L)	1.16	1.15	2.76↑	0.63	0.56	0.18	0.32	0.16	1.27	0.71	0.18	1.30
IgM (g/L)	1.07	0.86	1.23	2.32	1.36	0.72	1.99	0.77	1.51	1.23	0.37	1.84
IgE (KU/L)	21,705.67↑↑↑	13,472.28↑↑↑	2660.19↑↑	6336.65↑↑	5021.83↑↑	3709.67↑↑	9015.51↑↑	67.2	17,310.4↑↑↑	1841.29↑↑	43.87	221.65↑

The number in round bracket presents the age specific reference values according to Reference Values for Peripheral Blood Lymphocyte Subsets of Healthy Children in China

The reference value of IgG, IgA, IgM and IgE is 6.09–12.85 g/L, 0.52–2.16 g/L, 0.67–2.01 g/L and < 100 KU/L, respectively

Lymphocytes remained approximately normal in our patients, although the absolute numbers of total T cells (in P1 and P4), CD4 T cells (in P4 and P7) and total B cells (in P5 and P7) were slightly elevated. Four patients (P3, P7, P9, P10) had reduced numbers of NK cells (Table 2). We analyzed the T cell and B lymphocyte subpopulations in three patients who underwent blood sampling. All of these patients presented with elevated levels of double-negative T cells and reduced levels of memory B cells. P8 had a two-fold higher than normal level of effector memory cytotoxic T cells, while P9 had a twofold higher than normal level of terminally differentiated effector memory cytotoxic T cells. P4 had a dramatic reduction in γδ T cells (Table 3).

**Table 3:** Lymphocyte subpopulation of three STAT3 deficiency patient

	P4 (%)	P8(%)	P9 (%)
Cytotoxic T cell (CD8, CD45+CD3+CD8+)			
Central memory cytotoxic T cells (CD8 CM, CD3+CD8+CD45RA−CD27+)	10.40 (6.66–34.14)	19.20 (4.82–24.11)	9.00 (12.08–30.54)
Naive differentiated cytotoxic T cells (CD8 Naive, CD3+CD8+CD45RA+CD27+)	67.30 (38.19–86.18)	50.50 (47.36–92.45)	36.20 (41.58–77.90)
Effector memory cytotoxic T cells (CD8 EM, CD3+CD8+CD45RA−CD27−)	5.90 (0.60–12.01)	17.9↑ (0.20–8.94)	6.20 (1.58–13.18)
Terminally differentiated effector memory cytotoxic T cells (CD8 TEMRA, CD3+CD8+CD45RA+CD27−)	16.40 (0.50–24.45)	12.40 (0.15–28.32)	48.6↑ (1.70–24.62)
CD4 T cell (CD45+CD3+CD4+)			
Central memory helper T cells (CD4 CM, CD3+CD4+CD45RA−CD27+)	21.80 (17.12–47.60)	32.20 (10.15–33.38)	19.50 (22.06–46.46)
Naive differentiated helper T cells (CD4 Naive, CD3+CD4+CD45RA+CD27+)	75.80 (46.42–81.20)	65.40 (59.28–88.09)	76.30 (45.56–75.28)
Effector memory helper T cells (CD4 EM, CD3+CD4+CD45RA−CD27−)	2.30 (0.90–5.17)	2.00 (0.42–3.96)	4.10 (2.08–8.78)
Terminally differentiated effector memory helper T cells (CD4 TEMRA, CD3+CD4+CD45RA+CD27−)	0.00 (0.00–0.50)	0.40 (0.00–1.49)	0.10 (0.00–1.06)
γδ T cells (γδ T, CD3+TCRγδ+)	0↓ (5.07–17.60)	4.80 (3.95–10.40)	21 (6.92–19.84)
TCRαβ+ double-negative T (DNT) cells (CD3+TCRαβ+CD4−CD8−)	3.3↑ (0.56–2.36)	5.6↑ (0.41–1.55)	21.2↑ (0.18–2.81)
B cells (CD45+CD19+)			
Memory B (CD19+CD27+IgD−)	2.50↓ (3.60–18.55)	0.40↓ (1.77–7.06)	3.80↓ (7.76–19.90)
Naïve B (CD19+CD27–IgD+)	91.60↑ (59.59–85.28)	96.70↑ (75.28–92.77)	63.80 (48.36–75.84)
Transitional B (CD19+CD24++CD38++)	3.80 (4.73–15.68)	29.90↑ (6.04–21.62)	5.30 (2.58–12.30)
Plasmablasts (CD19+CD24−CD38++)	2.40 (0.60–10.31)	0.90 (0.71–5.88)	3.90 (0.90–7.36)

The number in round bracket presents the age specific reference values according to Reference Values for Peripheral Blood Lymphocyte Subsets of Healthy Children in China

### Nonimmunological abnormalities in 11 STAT3 LOF mutation patients

Two patients (2/11. P5 and P7) suffered from delayed growth. Retention of primary teeth also occurred in three patients (3/11. P1, P2 and P7). Three patients (3/11. P1, P5 and P7) developed the characteristic facial features. A broad nose and high-arched palate were observed in P1. Meanwhile, P5 and P7 had coarse facial skin and prominent foreheads. Scoliosis and pigeon breast occurred in only P7. P5 experienced a fracture of the collar bone. Cavernous hemangioma was observed in P9. Furthermore, lymphadenopathy of the neck and inguen was observed in 4 patients (P2, P4, P6 and P7). Splenomegaly was detected in P5 and P7, and the former also suffered from hepatomegaly. Furthermore, P5 and P10 exhibited food allergies (Fig. 1).

As previously reported [15], the National Institutes of Health (NIH) scoring system is the most commonly used clinical scoring system for STAT3-deficient diseases. In our study, 9 out of 11 patients reached or exceeded 40 points, and two patients scored below the diagnostic standard (P8 and P11, with scores of 27 and 21, respectively). We noticed that the evaluation year of P8 and P11 was 0.5 years and 0.83 years, respectively.

### STAT3 GOF mutation

P3 suffered from repeated cough, rhinitis, diarrhea and CMC. Diffusely enlarged lymph nodes and hepatosplenomegaly were observed in this patient. She also had autoimmune hemolytic anemia, reduced white blood cell and platelet counts, muscle weakness, diabetes, alopecia and delayed pubertal development. Her IgE level was 221.65 KU/L. The patient had severely reduced levels of all lymphocyte subsets. Her EBV-DNA viral load was 4.05E+04 in the PBMCs, and her mycoplasma viral load was 2.42E+08.

### Mutation of the STAT3 gene

**Fig. 2:**
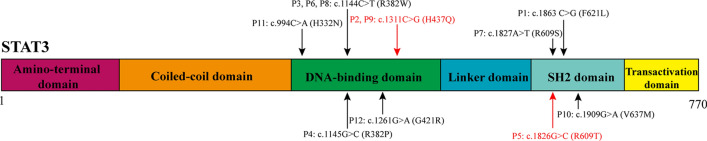
Schematic structure of STAT3 and mutation information in this study. STAT3 is a protein consisting of 770 amino acids. Six squares represent its protein domains, and all alterations in the base pairs discussed in this article are marked by arrows. The black arrows show the mutations that had been reported. The red arrows show the novel mutations in this study

**Fig. 3:**
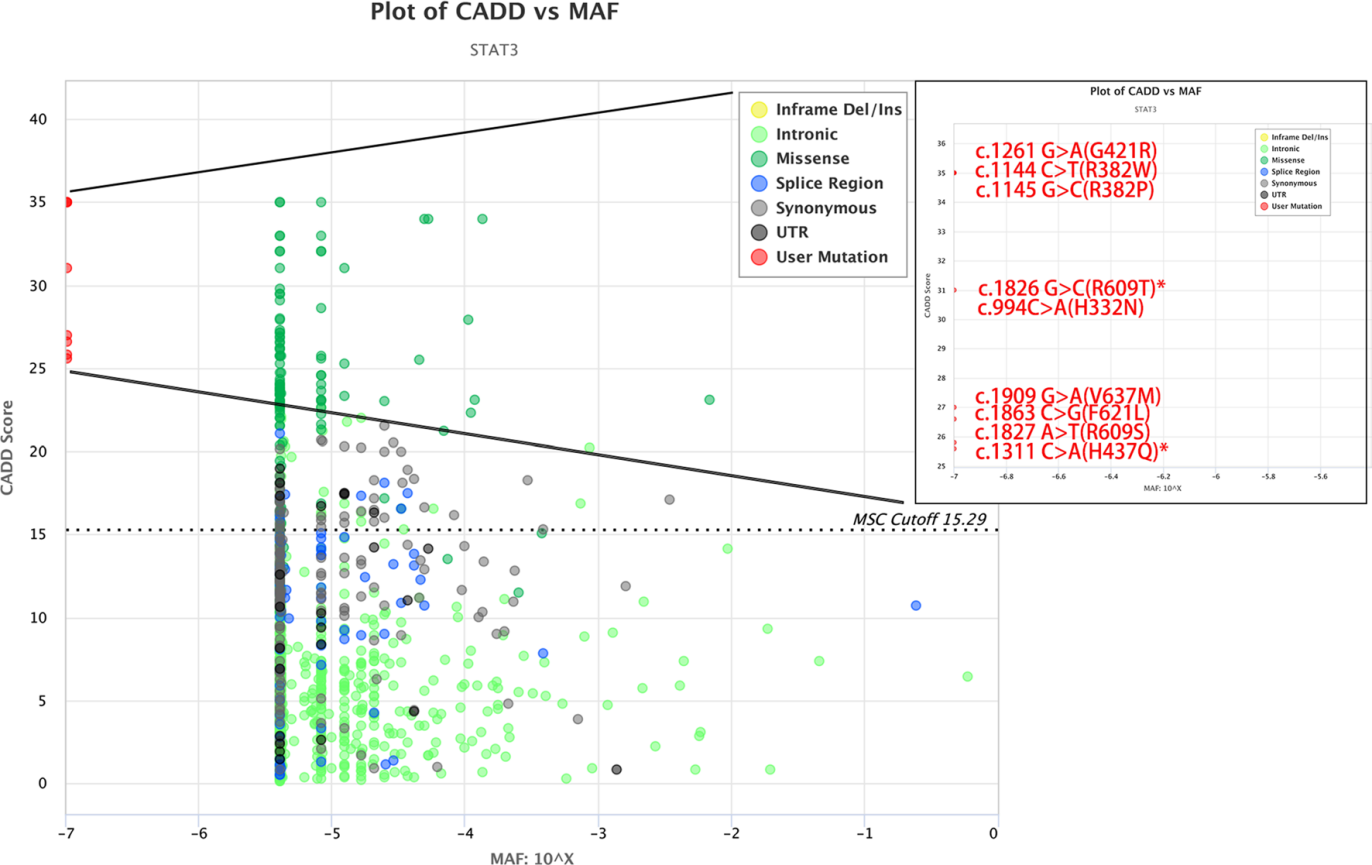
CADD vs. MAF plot of STAT3 by PopViz. The horizontal axis shows the MAF scores, and the vertical axis shows the CADD scores. The nine mutations in our patients (G421R, R382W, R382P, R609T, H332N, V637M, F621L, R609S and H437Q) are malignant and highlighted in red. All MAFs are − 7, and the CADD scores are 35, 35, 35, 31, 31, 27, 26.6, 25.8 and 25.6. *Shows the novel mutations in this study. CADD (Combined Annotation Dependent Depletion) score: Ranks genetic variants on the basis of diverse genomic features; MAF: minor allelic frequency in the Genome Aggregation Database (GnomAD)

WES suggested that these patients had heterozygous mutations in STAT3 (Fig. 2). As shown in Table 1, the variants were all de novo mutations except for the mutation in P11, who inherited the mutation from his father (c.994C>A; p.H332N). We noticed that c. 1144C>T (p.R382W) was the most common mutation in our study, which was identified in 3 patients (27.27%), followed by c. 1311C>A (p.H437Q) in 2 patients (18.18%). H437Q and R609T were two novel mutations that could not be found in the OMIM and ClinVar databases. Moreover, H437Q and R609T were predicted to be disease-causing mutations in MutationTaster; they were also predicted to be deleterious mutations by PopViz software [16] (Fig. 3). Moreover, the GOF mutation was a known mutation (1261G>A; p.G421R) that was proven to be GOF by Milner [17].

### Therapy

All 11 STAT3 LOF mutation patients were given prophylactic antimicrobials and symptomatic treatment. Eight patients (P1, P2, P3, P4, P5, P6, P8 and P11) achieved a notable improvement in their eczema, respiratory infections and candida infections after receiving intravenous immunoglobulin (IVIG) at a dose of 400–600 mg/kg/m^2^. Four patients (P1, P2, P4 and P6) underwent immunoglobulin monitoring after IVIG. The level of IgG remained normal or slightly increased. The STAT3 GOF mutation patient received treatment with the anti-IL6R monoclonal antibody tocilizumab and achieved a stable condition with less alopecia, normal blood glucose levels and fewer infections.

## Discussion

In this study, we retrospectively analyzed 12 patients with STAT3 mutations, all of whom were from unrelated parents. Most STAT3-deficient patients develop clinical presentations at an average age of 1.89 years. Our patients had a wide range in symptom onset age from neonatal to adolescence. Patients may be too young to present the classic phenotype of this disease. The diverse but generally young age of clinical onset makes the diagnosis of STAT3-HIES difficult. Our patients had an earlier age at initial diagnosis (4.74 years) than that reported in Chinese patients in 2017 (10.35 years) [11], which may be due to the increased awareness of STAT3-HIES and enhanced next-generation sequencing technology in recent years. We highlight the value of WES in diagnosing STAT3-HIES. Meanwhile, it is important to perform genetic counseling. Although most individuals develop STAT3-HIES owing to a de novo pathogenic mutation of STAT3, some patients inherit the disorder from a parent, such as our P11.

Furthermore, the most common clinical symptoms are respiratory and cutaneous infections, abscesses and fever. Likewise, consistent with previous reports [18, 19], we identified a high incidence of recurrent *Candida albicans* infections, which is associated with a damaged IL-17 and IL-22 response. However, limited by the real-world situation, we did not perform related functional experiments in the five patients who suffered from CMC. Additionally, there were no patients with *Aspergillus* infections, in contrast to the 17.5% of STAT3-deficient patients reported to experience aspergillosis in the lung cavities [20]. More than half of the patients developed otitis media, which is consistent with the reported percentage in a paper from China (47.06%) [21]. It had been described previously that gastrointestinal inflammation developed as a part of the manifestations of STAT3 LOF mutation [22]. However, diarrhea only occurred in 2 patients in our study. Congenital defects, such as atrial septal defects, laryngeal clefts and cryptorchidism, occurred to different degrees. In contrast to two large-scale STAT3-deficient cohort studies in China [11, 12, 23], few patients had the classic nonimmunological features, including particular facial features and dental and developmental abnormalities. This was a retrospective analysis; doctors are likely to overlook some slight facial changes, and many patients were young when they first visited our hospital. The lower incidence of primary teeth retention may be because they were young. The lack of scoliosis and osteoporosis in our patients can be explained by their young, pre-adolescent age.

We found that the increased IgE level was not entirely concomitant with the high eosinophil counts. STAT3 mutation has been reported to lead to impaired memory B cells [11, 12]. This is consistent with our finding that three patients had mildly damaged memory B cells. STAT3 plays a pivotal role in the development, differentiation and maintenance of T cell memory, and there are fewer central memory T cells in patients with HIES [8, 24]. The ability of CD8 T cells to control herpes viruses is reduced in STAT3-deficient patients, which may partly explain why a portion of our STAT3-deficient patients had EBV and CMV viremia [25]. The levels of naïve CD8 T cells were increased, while the levels of effector memory, central memory and terminally differentiated effector memory cytotoxic T cells were decreased in STAT3-deficient patients [26]. In contrast to a previous report, one patient in our study showed normal levels of central memory T cells but increased levels of effector memory T cells. Considering that STAT3-deficient patients have the ability to generate memory CD8 T cells [25], we speculate that the number of memory CD8 T cells may be increased, but their function may be impaired. Inconsistent with STAT3-deficient patients having a normal number of γδT cells [27], three of our patients had an obvious reduction in γδT cells. Not all patients had high NIH scores. The patients who did not meet the NIH score criteria for hyper IgE underwent evaluation before 1 year of age. We speculate that these two patients were too young to display a complete phenotype. Unfortunately, they have been lost to follow-up, making it impossible to repeat the evaluation at a later age.

A molecular study found that HIES is caused by variations in STAT3, Dedicator of cytokinesis 8 (DOCK8) [28], phosphoglucomutase 3 gene (PGM3) [9] and ZNF341 [29]. Simultaneously, STAT3-deficiency disease is categorized into two types: STAT3 LOF mutation disease comprises immunodeficiencies with syndromic features, while STAT3 GOF mutation disease involves immune dysregulation, especially regulatory T cell defects, according to the International Union of Immunological Societies in 2019 [30]. Our patients overwhelmingly had STAT3 LOF mutations. Regarding the different clinical presentations in STAT3 LOF and GOF mutation patients in our cohort, the latter had autoimmune disease, as mentioned in previous papers [17]. She also exhibited most of the traditional AD STAT3-deficient manifestations except eczema and highly elevated serum IgE levels.

We identified c. 1311C>A (p.H437Q) and c. 1826G>C (p.R609T), which are two novel STAT3 mutations in STAT3-deficient patients. Moreover, H437Q and R609T were predicated to be deleterious mutations by prediction software. The three patients carrying the novel mutations (P2, P5 and P9) had the clinical and immunologic features of STAT3 deficiency even though we did not perform functional experiments for those new mutation sites. Notably, one patient’s variant was inherited from his father, who had a de novo mutation in STAT3 and clinical symptoms that appeared when he was young. In keeping with numerous reports [11, 12, 23], c.1144C>T (p.R382W) is a hotspot mutation in patients carrying STAT3 gene mutations in China. On the other hand, in contrast to the literature, the second most common mutation, c. 1909G>A (p.V637M), was only identified in one patient in this study (9.09%). Genotype–phenotype correlations for STAT3 missense mutations have not been identified.

None of the patients died in our cohort. We speculate that the high survival rate is the result of the early multimodal treatment. We believe IVIG combined with antibiotic therapy may help reduce the opportunity for infection in STAT3-deficient patients. Moreover, it is important to administer therapy as soon as possible.

## Conclusion

In summary, this study is limited by the absence of long-term follow-up and related experimental validation. However, our research clearly illustrates the clinical, immunologic and genetic manifestations of STAT3-deficiency disease, and updates current knowledge by describing an earlier median age at diagnosis, a higher survival rate, and infrequent nonimmunological abnormalities. In addition, this report provided details regarding treatment, identified novel mutations and compared STAT3 LOF and GOF mutations in Han Chinese people. Ultimately, we extended the spectrum of STAT3-deficiency diseases in different ethnic groups.

## Data Availability

Not applicable.

## Abbreviations

AD: Autosomal dominant
BALF: Bronchoalveolar lavage fluid
CCDS: Consensus coding sequence
CMC: Chronic mucocutaneous candidiasis
CMV: Cytomegalovirus
DOCK8: Dedicator of cytokinesis 8
EBV: Epstein–Barr virus
GOF: Gain-of-function
HIES: Hyperimmunoglobulin E syndrome
HSCT: Hematopoietic stem cell transplantation
IgE: Immunoglobulin E
IVIG: Intravenous immunoglobulin
LOF: Loss-of-function
MRI: Magnetic Resonance Imaging
NIH: National Institutes of Health
NK: Natural killer
PBMCs: Peripheral blood mononuclear cells
PBS: Phosphate buffer solution
PGM3: Phosphoglucomutase 3 gene
STAT3: Signal transducer and activator of transcription 3
STAT3-HIES: STAT3-hyperimmunoglobulin E syndrome
VZV: Varicella–Zoster virus
WES: Whole genome sequencing

## References

[1] Mogensen TH (2018). IRF and STAT transcription factors—from basic biology to roles in infection, protective immunity, and primary immunodeficiencies. Front Immunol..

[2] Zhang Q, Boisson B, Beziat V, Puel A, Casanova JL (2018). Human hyper-IgE syndrome: singular or plural?. Mamm Genome.

[3] Vogel TP, Milner JD, Cooper MA (2015). The Ying and Yang of STAT3 in human disease. J Clin Immunol.

[4] Davis SD, Schaller J, Wedgwood RJ (1966). Job’s Syndrome. Recurrent, “cold”, staphylococcal abscesses. Lancet.

[5] Al-Shaikhly T, Ochs HD (2018). Hyper IgE syndromes: clinical and molecular characteristics. Immunol Cell Biol.

[6] Tavassoli M, Abolhassani H, Yazdani R, Ghadami M, Azizi G, Abdolrahim PHS (2019). The first cohort of iranian patients with hyper immunoglobulin e syndrome: a long-term follow-up and genetic analysis. Pediatr Allergy Immunol.

[7] Bergerson J, Freeman AF (2019). An update on syndromes with a Hyper-IgE phenotype. Immunol Allergy Clin North Am..

[8] Siegel AM, Heimall J, Freeman AF, Hsu AP, Brittain E, Brenchley JM (2011). A critical role for STAT3 transcription factor signaling in the development and maintenance of human T cell memory. Immunity.

[9] Hafsi W, Badri T (2020) Job syndrome (hyperimmunoglobulin E). In: StatPearls. StatPearls Publishing, Treasure Island, FL. https://www.ncbi.nlm.nih.gov/books/NBK525947. Accessed 10 Jan 2020.

[10] Fabre A, Marchal S, Barlogis V, Mari B, Barbry P, Rohrlich PS (2019). Clinical aspects of STAT3 gain-of-function germline mutations: a systematic review. J Allergy Clin Immunol Pract..

[11] Wu J, Chen J, Tian ZQ, Zhang H, Gong RL, Chen TX (2017). Clinical manifestations and genetic analysis of 17 patients with autosomal dominant hyper-IgE syndrome in Mainland China: new reports and a literature review. J Clin Immunol.

[12] Zhang LY, Tian W, Shu L, Jiang LP, Zhan YZ, Liu W (2013). Clinical features, STAT3 gene mutations and Th17 cell analysis in nine children with hyper-IgE syndrome in mainland China. Scand J Immunol.

[13] Dong X, Liu L, Wang Y, Yang X, Wang W, Lin L (2019). Novel heterogeneous mutation of TNFAIP3 in a Chinese patient with Behcet-like phenotype and persistent EBV viremia. J Clin Immunol.

[14] Ding Y, Zhou L, Xia Y, Wang W, Wang Y, Li L (2018). Reference values for peripheral blood lymphocyte subsets of healthy children in China. J Allergy Clin Immunol..

[15] Grimbacher B, Schäffer AA, Holland SM, Davis J, Gallin JI, Malech HL (1999). Genetic linkage of hyper-IgE syndrome to chromosome 4. Am J Hum Genet.

[16] Zhang P, Bigio B, Rapaport F, Zhang SY, Casanova JL, Abel L (2018). PopViz: a webserver for visualizing minor allele frequencies and damage prediction scores of human genetic variations. Bioinformatics.

[17] Milner JD, Vogel TP, Forbes L, Ma CA, Stray-Pedersen A, Niemela JE (2015). Early-onset lymphoproliferation and autoimmunity caused by germline STAT3 gain-of-function mutations. Blood.

[18] Hiller J, Hagl B, Effner R, Puel A, Schaller M, Mascher B (2018). STAT1 gain-of-function and dominant negative STAT3 mutations impair IL-17 and IL-22 immunity associated with CMC. J Invest Dermatol..

[19] Puel A, Cypowyj S, Marodi L, Abel L, Picard C, Casanova JL (2012). Inborn errors of human IL-17 immunity underlie chronic mucocutaneous candidiasis. Curr Opin Allergy Clin Immunol.

[20] Dureault A, Tcherakian C, Poiree S, Catherinot E, Danion F, Jouvion G (2019). Spectrum of pulmonary aspergillosis in hyper-IgE syndrome with autosomal-dominant STAT3 deficiency. J Allergy Clin Immunol Pract..

[21] Wu J, Hong L, Chen TX (2018). Clinical manifestation of hyper IgE syndrome including otitis media. Curr Allergy Asthma Rep..

[22] Arora M, Bagi P, Strongin A, Heimall J, Zhao X, Lawrence MG (2017). Gastrointestinal manifestations of STAT3-deficient hyper-IgE syndrome. J Clin Immunol.

[23] Deng Y, Li T, Xie X, Xia D, Ding L, Xiang H (2019). Hyper IgE syndrome associated with novel and recurrent STAT3 mutations: two case reports. Medicine..

[24] Deenick EK, Pelham SJ, Kane A, Ma CS (2018). Signal transducer and activator of transcription 3 control of human T and B cell responses. Front Immunol..

[25] Kane A, Deenick EK, Ma CS, Cook MC, Uzel G, Tangye SG (2014). STAT3 is a central regulator of lymphocyte differentiation and function. Curr Opin Immunol.

[26] Ives ML, Ma CS, Palendira U, Chan A, Bustamante J, Boisson-Dupuis S (2013). Signal transducer and activator of transcription 3 (STAT3) mutations underlying autosomal dominant hyper-IgE syndrome impair human CD8(+) T-cell memory formation and function. J Allergy Clin Immunol..

[27] Wilson RP, Ives ML, Rao G, Lau A, Payne K, Kobayashi M (2015). STAT3 is a critical cell-intrinsic regulator of human unconventional T cell numbers and function. J Exp Med.

[28] Biggs CM, Keles S, Chatila TA (2017). DOCK8 deficiency: insights into pathophysiology, clinical features and management. Clin Immunol..

[29] Beziat V, Li J, Lin JX, Ma CS, Li P, Bousfiha A (2018). A recessive form of hyper-IgE syndrome by disruption of ZNF341-dependent STAT3 transcription and activity. Sci Immunol..

[30] Tangye SG, Al-Herz W, Bousfiha A, Chatila T, Cunningham-Rundles C, Etzioni A (2020). Human inborn errors of immunity: 2019 update on the classification from the international union of immunological societies expert committee. J Clin Immunol.

